# Novel Corrugated Long Period Grating Surface Balloon-Shaped Heterocore-Structured Plastic Optical Fibre Sensor for Microalgal Bioethanol Production

**DOI:** 10.3390/s23031644

**Published:** 2023-02-02

**Authors:** Sanober Farheen Memon, Ruoning Wang, Bob Strunz, Bhawani Shankar Chowdhry, J. Tony Pembroke, Elfed Lewis

**Affiliations:** 1 Optical Fibre Sensors Research Centre, University of Limerick, V94 T9PX Limerick, Ireland; 2 Department of Electronic and Computer Engineering, University of Limerick, V94 T9PX Limerick, Ireland; 3 Key Laboratory of In-Fiber Integrated Optics of Ministry of Education, College of Physics and Optoelectronic Engineering, Harbin Engineering University, Harbin 150001, China; 4 NCRA-CMS Lab, IICT, Mehran University of Engineering and Technology, Jamshoro 76062, Sindh, Pakistan; 5 Department of Chemical Sciences and Bernal Institute, University of Limerick, V94 T9PX Limerick, Ireland

**Keywords:** heterocore structure, plastic optical fibre, long period grating, macro bending, ethanol sensor, bioethanol production using microalgae

## Abstract

A novel long period grating (LPG) inscribed balloon-shaped heterocore-structured plastic optical fibre (POF) sensor is described and experimentally demonstrated for real-time measurement of the ultra-low concentrations of ethanol in microalgal bioethanol production applications. The heterocore structure is established by coupling a 250 μm core diameter POF between two 1000 μm diameter POFs, thus representing a large core—small core—large core configuration. Before coupling as a heterocore structure, the sensing region or small core fibre (SCF; i.e., 250 μm POF) is modified by polishing, LPG inscription, and macro bending into a balloon shape to enhance the sensitivity of the sensor. The sensor was characterized for ethanol–water solutions in the ethanol concentration ranges of 20 to 80 *%v/v*, 1 to 10 *%v/v*, 0.1 to 1 *%v/v*, and 0.00633 to 0.0633 *%v/v* demonstrating a maximum sensitivity of 3 × 10^6^ %/RIU, a resolution of 7.9 × 10^−6^ RIU, and a limit of detection (LOD) of 9.7 × 10^−6^ RIU. The experimental results are included for the intended application of bioethanol production using microalgae. The characterization was performed in the ultra-low-level ethanol concentration range, i.e., 0.00633 to 0.03165 *%v/v*, that is present in real culturing and production conditions, e.g., ethanol-producing blue-green microalgae mixtures. The sensor demonstrated a maximum sensitivity of 210,632.8 %T/*%v/v* (or 5 × 10^6^ %/RIU as referenced from the RI values of ethanol–water solutions), resolution of 2 × 10^−4^ *%v/v* (or 9.4 × 10^−6^ RIU), and LOD of 4.9 × 10^−4^ *%v/v* (or 2.3 × 10^−5^ RIU). Additionally, the response and recovery times of the sensor were investigated in the case of measurement in the air and the ethanol-microalgae mixtures. The experimentally verified, extremely high sensitivity and resolution and very low LOD corresponding to the initial rate of bioethanol production using microalgae of this sensor design, combined with ease of fabrication, low cost, and wide measurement range, makes it a promising candidate to be incorporated into the bioethanol production industry as a real-time sensing solution as well as in other ethanol sensing and/or RI sensing applications.

## 1. Introduction

**Figure 1:**
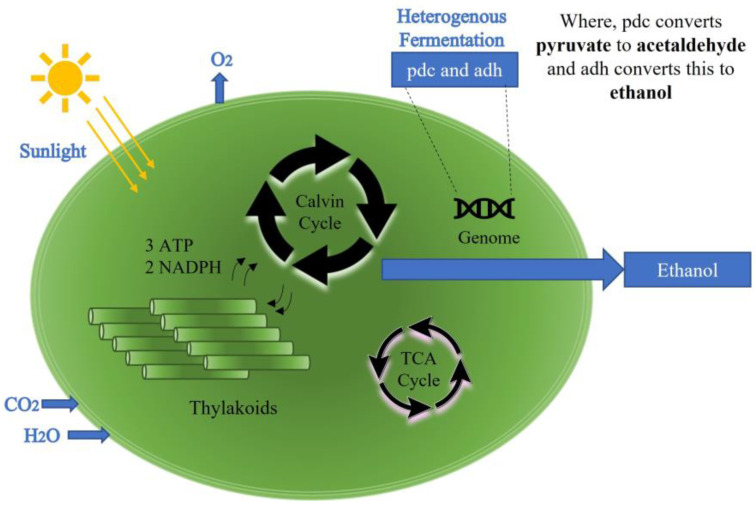
Chemical process and integration of the pdc/adh cassette into the Synechocystis PCC6803 model. Adapted from [7].

As the global supply of fossil fuels diminishes and the demand for liquid fuels to run transportation increases, there has been a push to find alternative fuels. One option is ethanol as a renewable fuel or a partial substitute for gasoline. Although ethanol can be produced chemically, the production process and the materials required for its synthesis are not sustainable. Hence, scientists have explored various biological alternative sources for ethanol production. Bioethanol can be produced using various types of biomass such as edible crops, woody plants, and algal biomass [1] and is therefore often classified according to its derivation from these sources. First generation biofuels refer to biofuels that were produced using food-based or edible sources such as vegetable oils, high starch, and high carbohydrate crops. Biofuels derived from non-food plants are classed as second generation biofuels. Algae are the biomass source for third generation biofuels [1]. Ethanol as a biofuel is considered a low-carbon alternative to fossil fuels. The wider usage of edible and woody crops as substrates is widely considered unsustainable and could have additional environmental impacts, e.g., marginalization of potential food-producing agricultural spaces [2]. The current and fourth generation of biofuels relies on the metabolic engineering of algal biomass (which needs to be engineered to convert the substrate to ethanol), which is considered to be a more sustainable approach [3]. Cyanobacteria, also known as microalgae, have demonstrated potential to produce ethanol through metabolic engineering and have relatively modest growth requirements i.e., minerals and salts (which can be supplied experimentally by BG11 media, or by brackish river water in a large-scale operation). BG11 broth is a universal nutritional medium for the growth and cultivation of blue-green algae which derive their energy from sunlight via photosynthesis similar to plants and obtain all their carbon from atmospheric or dissolved carbon dioxide (CO_2_). Thus, they are environmentally sustainable and can contribute to atmospheric and water-based CO_2_ reduction. The production of ethanol can be achieved using the experimental model cyanobacterium *Synechocystis* PCC6803, which has been genetically engineered to produce ethanol [4]. The authors of this article have implemented this by introducing two genes encoding pyruvate decarboxylase (pdc) which converts the central metabolic intermediate pyruvic acid to acetaldehyde and alcohol dehydrogenase (adh) which in turn converts acetaldehyde to ethanol. In this process, the standard *Synechocystis* PCC6803 is converted into an alcohol producer *Synechocystis* UL004 [5,6]. The main chemical process and integration of the pdc/adh cassette into the *Synechocystis* PCC6803 model is presented schematically in Figure 1, which is adapted from [7].

One key aspect of the production was the ability to instantaneously measure ethanol productivity during each phase of the microbial production process to ensure that the production organism is active and the production conditions are optimal. These include monitoring optimal light conditions and temperature, monitoring for the presence of inhibitory substances, and providing the correct amount of CO_2_. Additionally, the initial rate of production of ethanol using the cyanobacterium *Synechocystis* UL004 was defined as 0.1–0.5 g·L^−1^ per day [4]. This rate corresponds to %volume/volume (*%v/v*) concentration as 0.0126 to 0.0633 *%v/v*. This in turn corresponds to minute changes in the refractive index of an ethanol-producing microalgal suspension or mixture. Hence, a highly sensitive and in-line measuring method for monitoring ethanol productivity is an essential element for process optimization, particularly for process scale-up. The concentration of ethanol in an aqueous solution can be measured using several techniques. Raman spectroscopy [8], enzymatic measurement [9], UV/NIR spectroscopy [10], dichromatic oxidation spectrophotometry [11], refractive index (RI) analysis [10], high-performance liquid chromatography (HPLC) [12], gas chromatography (GC) [13,14], capillary electrophoresis [15], pycnometry [16], densimetry [16], hydrometry [16], and colorimetric methods [17] are some of the most commonly used techniques. However, there are several disadvantages associated with using these approaches, including the possibility of sample loss, low reproducibility, long analysis time, complicated offline sample preparation processes, and heavy and costly analytical instrumentation.

Plastic optical fibre (POF) sensors have demonstrated significant advancement in chemical sensing compared with the techniques mentioned above, due to their versatility, real-time and remote deployment capability, ruggedness, cost-effectiveness, ability to be miniaturized, and ease of integration with electronic or optoelectronic systems. Hence, POFs represent a potential solution for the high sensitivity requirement of the in-line measurement of ethanol during its production using microalgae. A variety of optical fibre sensing methods have been reported to measure ethanol in various applications including those based on absorption, interferometric, fibre grating, and plasmonic [18]. Most optical fibre ethanol sensors are based on glass optical fibres (GOFs); however, the fabrication of sensors using GOFs can often be complicated and requires specialized instruments. For example, fibre Bragg grating (FBG)- and long-period grating (LPG)-based sensors require complicated and expensive photolithographic or femtosecond laser writing processes, wavelength modulation-based interferometric sensors require delicate and expensive interrogation systems, and absorbance-based sensors exhibit fragility due to the deformation of the fibres.

POFs have additional features of flexibility, ruggedness, ease of fabrication/handling, low cost, and operation in the visible range. These attributes have attracted significant attention and make them an ideal candidate for developing low-cost and robust ethanol sensors for industrial use. Different types of POF-based sensors have been proposed to measure ethanol concentration. Examples include an intensity-based tapered sensor coated with a mixture of polyvinyl alcohol (PVA) and SiO_2_ [19], a U-bend sensor for ultra-low level ethanol concentration measurement [20,21], a carbon nanotube (CNT) and graphene oxide (GO)-coated unclad sensor [22], a localized surface plasmon resonance (LSPR) based U-bent sensor [23], and a D-shaped surface plasmon resonance (SPR) sensor [24]. However, these sensors were either characterized only for ethanol-water solutions or intended for high-level ethanol concentration measurements, e.g., in the 20% to 100% range. For the application reported in this article, higher sensitivity and the need for avoiding cross-sensitivity from other algae-produced chemicals are deemed high priority requirements, which will require a highly sensitive and robust sensor design.

Fibre LPG-based sensors are also attracting significant interest for refractive index measurements due to their high sensitivity and ease of fabrication, and have generally been implemented using GOFs [25,26]. Heterocore-structured optical fibre sensors have also been explored for refractive index measurements using GOFs [27,28,29] and to the best of the authors’ knowledge, the concept of using POFs for heterocore-structured sensors remains a unique and highly innovative approach.

In this work, the POFs of two different diameters are used in a balloon-shaped heterocore structure, representing a large core—small core—large core design. An LPG was inscribed on the polished surface of the thin core POF of the heterocore structure in order to enhance the sensitivity for the measurement of ethanol in the microalgal mixtures. It is a unique sensor design combining the corrugated surface LPG, macro-bending, and heterocore structure using POF, achieving ultra-high sensitivity, i.e., 3 × 10^6^ %/RIU for measurement of ethanol concentration in water and 210,632.8 %Transmittance/*%v/v* (or 5 × 10^6^ %/RIU) for ethanol production in the microalgal mixture. In this investigation the sensor was characterized for both ethanol–water solutions and in ethanol–microalgae mixtures at a range even lower than the initial rate of ethanol production using *Synechocystis* UL004, i.e., as low as 0.00633 *%v/v* (%volume/volume concentration). This novel sensor design demonstrated repeatable real-time measurements of ultra-low-level ethanol concentration changes in water and in the presence of ethanol-producing microalgae, combined with ease of fabrication, low cost, ruggedness, and excellent response time, thus providing great potential for measurement in industrial ethanol production applications. The sensor was also characterized for higher ranges of ethanol in water (i.e., 0.1 to 1 *%v/v*, 1 to 10 *%v/v*, and 20 to 80 %*v*/*v*). Operation in these higher measurement ranges makes it also a promising candidate for other ethanol sensing applications.

## 2. Sensing Mechanism

**Figure 2:**
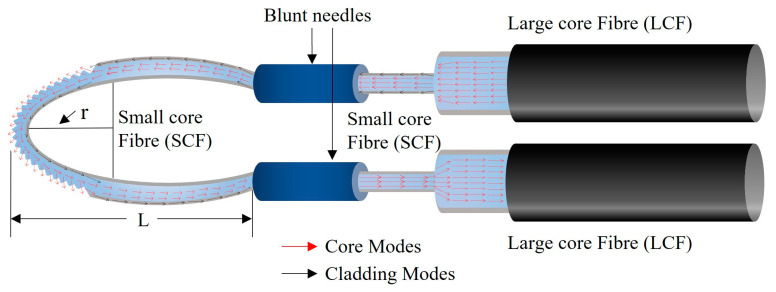
Schematic of the corrugated LPG surface, balloon-shaped heterocore-structured POF sensor.

A schematic of the corrugated LPG surface, heterocore-structured, balloon-shaped POF sensor is shown in Figure 2. The sensor consists of two large core multimode POF and one small core multimode POF. The small core fibre (SCF) is inserted between two large core fibres (LCFs), forming a large core—small core—large core heterocore structure adapted from a classical multimode—single mode—multimode (MSM) heterocore structure. The SCF provides the sensing region and the two LCFs serve as input and output light waveguides to the SCF. In addition to the light coupling in the core of the SCF, a significant part of the coupled light power also leaks into the cladding of the SCF at the heterocore structure’s input interface due to the significant difference in the diameters of the LCFs and SCF. The large number of additional cladding modes generate a significant evanescent field at the interface of the cladding of the SCF and the surrounding medium.

A small part of the SCF was polished to enhance the sensitivity of the sensing region, and an LPG was subsequently inscribed. The LPG was inscribed by application of pressure to the fibre using a specially designed mechanical compression jig which produced a linear V-grooved array with a pitch of 100 µm. The LPG-inscribed SCF was then coupled to the two LCFs in a balloon-shaped geometry to enhance more cladding modes and produce a stronger evanescent field interaction at the SCF external boundary. This is because macro bending of the fibre enhances cladding modes and introduces propagation losses due to the increase in total internal reflection (TIR) events in the fibre as well as the penetration depth of the evanescent field into the surrounding medium. This, in turn, enhances the light interaction with the surrounding medium. The length and bend radius of the balloon-shaped structure are defined as L and r.

As seen in Figure 2, the diameter of the LCF is significantly larger than the SCF, hence, some of the coupled light from the input LCF leaks into the cladding of SCF and creates two different paths: one in the core and the other in the cladding of SCF as it would in a classical MSM structure [29]. Due to the larger number of core modes in the multimode POF used in this investigation, a clear interference pattern was not established. However, cladding modes leak into the surrounding environment or solution following interaction with the LPG region. According to the working principle of an LPG, optical coupling occurs in the grating region between the forward propagating core mode and the cladding modes at certain wavelengths *λ*. The resonances of the LPG are determined by the phase matching condition as given below in Equation (1):

(1) $$\lambda =\Lambda \left(n_{eff}^{co}-n_{eff}^{cl}\right)$$

where *Λ* is the grating period and $n_{eff}^{co}$ and $n_{eff}^{cl}$ are the effective refractive indices in the core and cladding, respectively. This applies to the single-mode fibres where only one core mode exists and it only couples with cladding modes at certain wavelengths. The large number of core and cladding modes in a multimode fibre leads to the coupling of multiple core modes with higher order, cladding, and radiation modes. Hence, POF-based LPG sensors are suitable for intensity modulation schemes and wavelength dependence is not supported. However, the leaky modes around the LPG region produce a significant evanescent field. Thereafter, the light propagating through the bent area couples back into the output LCF.

Therefore, the sensor’s operating principle is based on the evanescent field and propagation losses introduced by the heterocore structure, balloon shape, and LPG inscription on a polished surface. The changing surrounding refractive indices (RIs) modulate the sensor’s response.

## 3. Materials and Methods

### 3.1. Materials

The fibres used throughout the sensor were commercial POFs (Asahi Kasei Corporation, Tokyo, Japan) with a polymethyl methacrylate (PMMA) core and fluorinated polymer cladding, sourced from FiberFin, Inc., Yorkville, IL, USA. The heterocore structure was fabricated by mechanically coupling the 250 μm diameter POF (i.e., SCF) between two 1000 μm POF (i.e., LCF). The parameters of the POFs are listed in Table 1.

**Figure 3:**
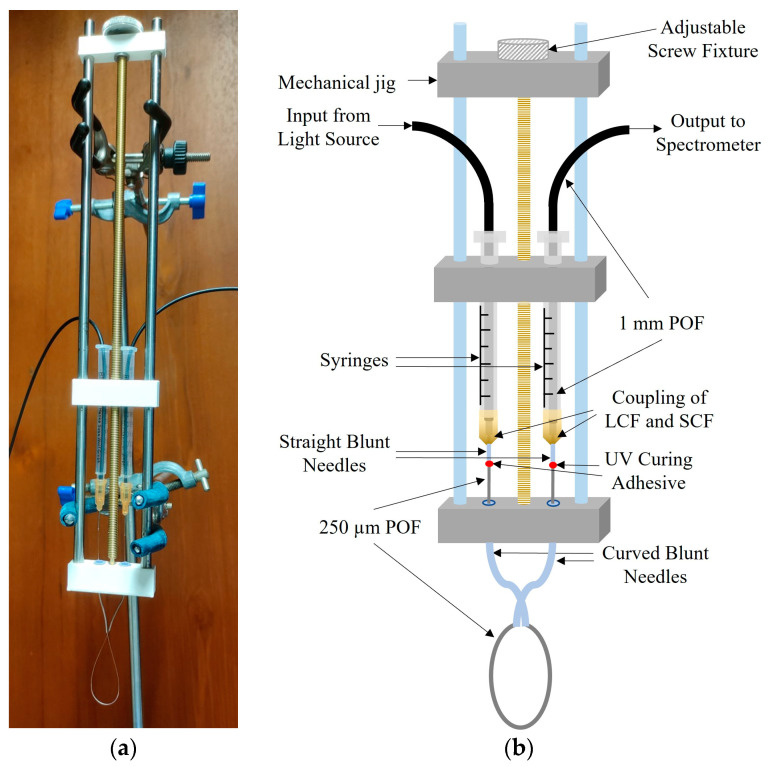
Arrangement of the LPG-inscribed, balloon-shaped heterocore-structured sensor using an in-house designed mechanical jig. (**a**) Photograph; (**b**) schematic representation.

A dedicated mechanical jig was designed in-house to mechanically couple the POFs. A photograph of the sensor arrangement with the jig is presented in Figure 3a and its schematic representation is in Figure 3b. The fibres were brought together within the two syringes with straight blunt needles on either side of an adjustable screw fixture similar to a single-axis manual translational stage. The syringes with straight blunt needles were used to insert and fix the position of the LCFs whilst maintaining the two ends of the SCF in the correct coupling position. The curved blunt needles at the end of the jig were used to insert the two ends of SCF for creating a balloon shape and then two ends of the SCF were coupled to the LCFs passing through the straight blunt needles, where both ends of the SCF were fixed using UV curing adhesive (Thorlabs NOA68—Norland Optical Adhesive). The adjustable screw fixture was used to adjust the radius of the balloon section of the sensor. This jig allowed the optimum configuration of the sensor to be easily determined using simple adjustments whilst maintaining the robustness and rigidity of the test setup. The sensor setup was tested for robustness against fibre movement and mechanical vibration.

A 99.8% Fisher Ethanol (C_2_H_5_OH) reagent was used for the ethanol–water solution and ethanol–microalgae mixture experiments. For characterization of the sensor for ethanol sensing in the microalgal mixtures, a mixture of distilled water, BG11 broth (Sigma Aldrich, St. Louis, MO, USA—Lot: BCBT9729) and Cyanobacterium *Synechocystis* PCC6803 (stored in the form of concentrated freeze-dried powder at −80 °C) was used. The recipe for the mixture is as follows:

1. To make the BG11 solution, add 10 mL of concentrated BG11 Broth to 990 mL of distilled water and mix well.
2. Divide the prepared solution in half and add the freeze-dried *Synechocystis* concentrate to one half. Reserve half of the BG11 solution to adjust the concentration of the microalgae mixture.
3. Shake well to dissolve.

### 3.2. Sensor Fabrication

Two equal lengths, i.e., 90 cm, of LCFs were cut and a small part of the jacket was removed from both ends of LCFs. Both ends of the LCFs were polished to ensure smooth coupling with the light source and spectrometer and the opposite ends with the SCF. The prepared LCF samples were fixed in the syringes with straight blunt needles attached to the designed mechanical test jig as described above.

**Figure 4:**
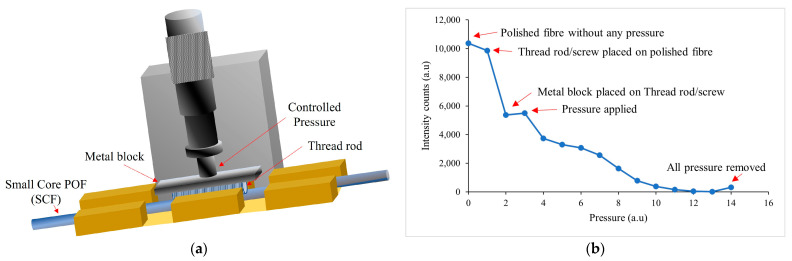
LPG fabrication, (**a**) LPG fabrication system. (**b**) Decreasing spectrum intensity at 694 nm wavelength, monitored during LPG fabrication.

The sensing region or SCF was prepared using an unjacketed 250 μm diameter POF. A small piece of SCF was cut and its ends were polished manually on a flat bed with a 2000-grit sanding paper (RS PRO P2000 grit sanding sheet) and a 3 µm polishing film (FIS L12F3 3 µm Aluminium Oxide polishing film) in a standard format. The SCF was then fixed on a flat surface for side polishing. A 30 mm portion in the middle of the fibre was polished for 1 min and 15 s using a rough sanding sheet (RS PRO P2000 grit sanding sheet) and then fine polished for 15 s using a fine polish film (FIS L12F3 3 µm Aluminium Oxide polishing film). After polishing, the LPG was fabricated using the die-press print method as demonstrated in Figure 4a. The polished SCF sample was placed in an in-house-designed fibre holder in a straight position and coupled to the LCFs at both ends to monitor the LPG fabrication process and avoid breaking the fibre due to excessive pressure. A commercially available thread rod (Micro-positioning Screw with 254 threads per inch (TPI) from Kozak Micro Adjusters, Randolph, NJ, USA) was placed over the fibre and a metal block was placed over the thread rod. Controlled pressure was applied on the metal block using a translational stage to deform the fibre to form a mould of the thread rod. The LPG fabrication process was monitored and the experimental layout and typical signal progression for this are shown in Figure 4b including all the steps of applying and removing pressure. The monitoring was performed using the full spectrum of the tungsten halogen light source (Ocean Optics LS-1 Series). However, for the purpose of clarity, a single wavelength was selected, i.e., 694 nm, and the intensity signal at that value was monitored. It can be seen that, with increasing pressure, the intensity of the transmitted light signal decreased.

**Figure 5:**
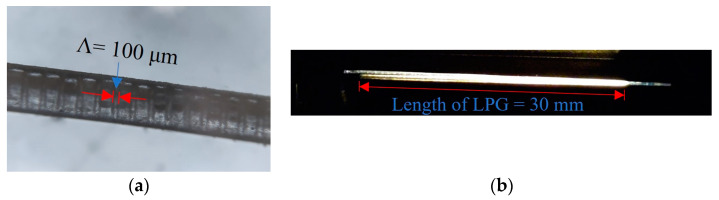
LPG images. (**a**) Microscope image of LPG on the small core (250 μm) POF. (**b**) Photo of LPG showing the light leakage from the corrugated LPG surface.

An LPG with a 100 µm pitch/period (Λ) was inscribed and a microscope image of the formed LPG is shown in Figure 5a. Figure 5b shows the fabricated LPG irradiated using the tungsten halogen lamp light source, showing the leakage of propagating light from the corrugated LPG surface. The LPG-inscribed SCF was then inserted through the curved blunt needles in the jig and coupled to the LCFs, creating a balloon shape as shown in Figure 3. The SCF was fixed at the point of insertion in the straight blunt needles using UV curing glue. The resulting length (L) and bending radius (r) of the balloon shape structure were 4.2 cm and 1 cm, respectively.

**Figure 6:**
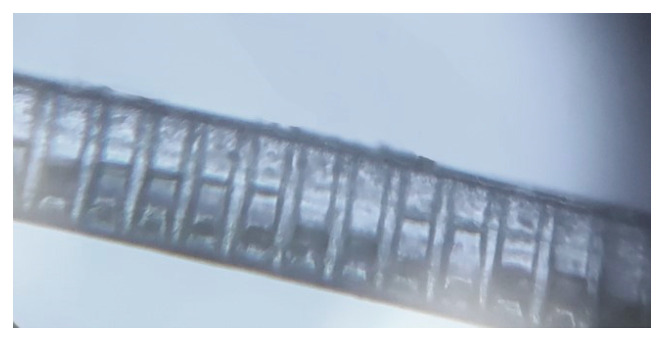
Microscope image of the LPG on the small core (250 μm) POF following a non-use period of three months.

The LPG-inscribed POF was placed in a clean, sealed enclosure for over three months immediately following experimentation in order to observe any possibility of a recovery effect, i.e., deformation or creep of the LPG and polymer material. Figure 6 is a microscope image of the LPG after three months and it is clear that there is no evidence of LPG deformation on the SCF, i.e., the sensor is mechanically stable with time and no recovery effect was observed.

**Figure 7:**
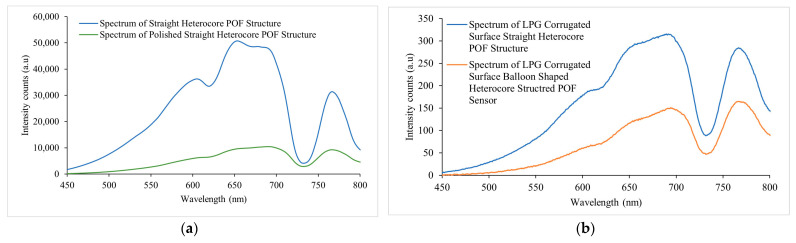
Measured spectra during the fabrication stages of the corrugated LPG surface, balloon-shaped heterocore-structured POF sensor in an air medium. (**a**) Spectrum of straight heterocore POF structure without polishing and LPG on the SCF and spectrum of straight heterocore POF structure following polishing on the SCF. (**b**) Spectum of straight and balloon-shaped heterocore POF structure with polishing and LPG on the SCF.

The output spectra of the sensor were acquired in the wavelength range of 450 nm to 800 nm at each step of fabrication in the air medium and illustrated in Figure 7a, i.e., the spectra of straight heterocore structure and polished surface straight heterocore structure. Figure 7b shows the spectra of the LPG corrugated surface, straight heterocore structure, and LPG-corrugated surface, balloon-shaped heterocore structure. It can be seen that with each step, the intensity of the transmission spectrum decreased as more light leaked from the prepared surface of the SCF due to polishing, LPG engraving, and macro bending. There was a greater intensity decrease from 600 nm to 700 nm in comparison to the peak in the range of 750 nm to 800 nm as can be seen when comparing with the spectra in Figure 7a, which showed a percentage intensity variation of 128% and 109% measured at 691.45 nm and 766.88 nm, respectively.

**Figure 8:**
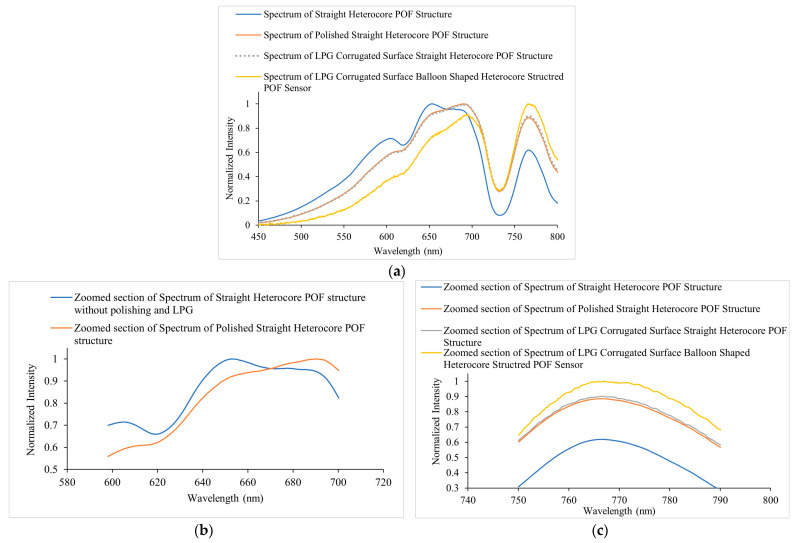
(**a**) Normalized intensity spectra during the fabrication stages of corrugated LPG surface balloon-shaped heterocore-structured POF Sensor in air medium. (**b**) Zoomed section of the spectra of Straight heterocore POF structure and polished straight heterocore POF structure in the 600 nm to 700 nm region. (**c**) Zoomed section of spectra of straight heterocore POF structure, polished straight heterocore POF structure, straight and balloon-shaped heterocore POF structure with polishing and LPG in the 750 nm to 790 nm region.

Figure 8a represents the normalized intensity graphs for the spectra in Figure 7a,b for comparing the spectral changes in the sensor’s response. A visible change in the spectral peaks was evident within the 600 nm to 700 nm wavelength range when compared with the spectra for the POF heterocore structure with and without side polishing and the presence of the LPG, indicating a more pronounced peak in the 675 nm to 700 nm wavelength range and diminishing peaks in the 600 nm to 675 nm wavelength region. This was demonstrated more clearly in the zoomed section of the spectra of the straight heterocore structure and polished surface straight heterocore structure in the 600 nm to 700 nm wavelength region in Figure 8b. A relative intensity increase was also seen for the peaks in the 750 nm to 800 nm wavelength range progressing with the modification of the sensor (i.e., polishing, LPG, and macro bending/balloon shape) as demonstrated more prominently in the zoomed section of all the spectra in the 750 nm to 790 nm wavelength range in Figure 8c. These spectral changes can be explained as being due to the optical coupling of higher order modes out of the fibre core in the case of the forward propagating core modes in the 675 nm to 700 nm and 750 nm to 800 nm wavelength ranges and the loss of forward propagating core modes as radiation or leaky modes in the 600 nm to 675 nm wavelength range at the polished and LPG-inscribed surfaces of the SCF.

### 3.3. Experimental Setup and Sensor Calibration

**Figure 9:**
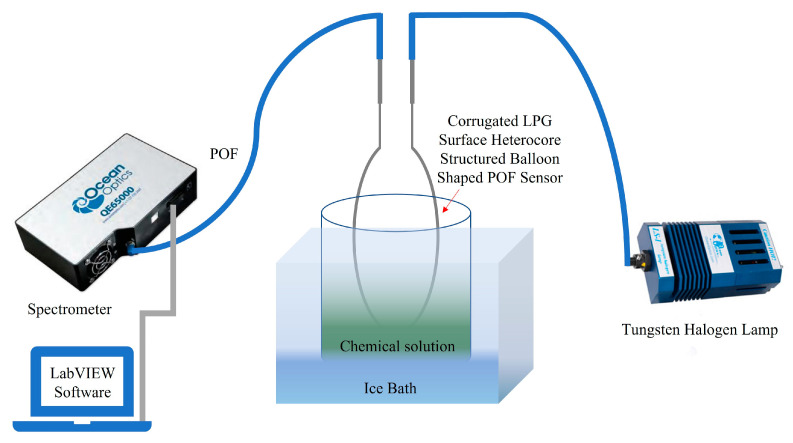
Illustration of the experimental setup.

Figure 9 shows the schematic of the experimental setup that was used to measure the output of the sensor in the ethanol–water solutions and ethanol–microalgae mixtures. A tungsten halogen broadband light source (Ocean Optics LS-1 Series) was used as the light source. The light source spectrum remained stable when monitored for its stability over a period of 30 min to rule out the influence of input power fluctuations during measurements. A scientific-grade spectrometer (Ocean Optics QE65000) was used to detect the output from the sensor using an integration time of 100 ms and a PC-based LabVIEW program was used to acquire and save the data from the spectrometer in real time. Ethanol is a volatile compound and evaporates at normal room temperature. Therefore, an ice bath was used to maintain the temperature of the solutions between 1 °C and 4 °C during the measurements. All experiments were performed in a dark room at a constant temperature of 1 °C to 4 °C and the temperature was monitored to observe any fluctuations.

**Figure 10:**
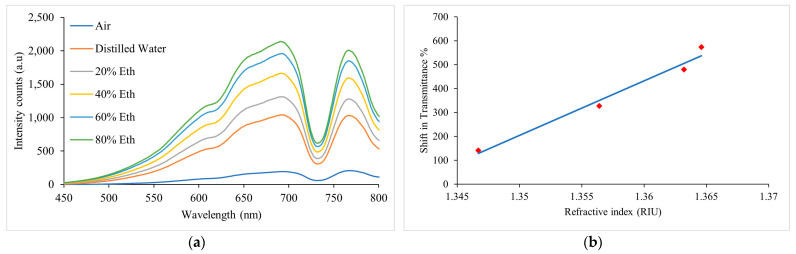
Sensor calibration. (**a**) Spectral responses of the sensor for air, water, and ethanol–water solutions at 20 %*v/v*, 40 %*v/v*, 60 %*v/v*, and 80 %*v/v* concentration in the wavelength range of 450 nm to 800 nm. (**b**) Sensor’s calibration curve for the RIs of ethanol–water solutions at 20 %*v/v*, 40 %*v/v*, 60 %*v/v*, and 80 %*v/v* concentration at wavelength of 691.45 nm.

Calibration of the sensor was performed by characterizing the sensor for air, distilled water, and 20 to 80 *%v/v* ethanol in the ethanol-water solutions. Prior to calibration, solutions of distilled water and ethanol, with an ethanol concentration of 20 %*v/v*, 40 %*v/v*, 60 %*v/v*, and 80 %*v/v* were prepared. The refractive indices (RIs) of these solutions were measured using an Abbe Refractometer operating at 20 °C. Figure 10a shows the spectral responses of the sensor for ethanol–water solutions at different concentrations in the wavelength range of 450 nm to 800 nm. It can be seen that with increasing RI or ethanol percentage in the solution, transmission intensity increased. Figure 10b shows the sensor response representing the shift in transmittance % as a function of the RIs of the ethanol–water solutions corresponding to ethanol concentrations of 20 %*v/v*, 40 %*v/v*, 60 %*v/v*, and 80 %*v/v* in distilled water, i.e., 1.3467, 1.3564, 1.3632, and 1.3646, respectively, at a wavelength of 691.45 nm where the maximum intensity peak was identified in the spectrum. The transmittance % (*T*%) can be expressed as:

(2) $$T\%=\frac{I_{n}}{I_{o}}\times 100$$

where $I_{n}$ and $I_{o}$ are the intensity counts in the RI measurements of the ethanol–water solutions and air, respectively. The established calibration curve for the sensor is described by:

(3) ΔT%=22,806 n−30,583

where ΔT% represents the shift in transmittance % and *n* is the RI of the ethanol–water solution.

## 4. Experimental Results

### 4.1. Analysis of Ethanol Sensing in Water

The sensor characterization was performed in real-time and using the experimental setup and conditions described in Section 3. The initial rate of bioethanol production using *Synechocystis* UL004 is known to be between 0.1 and 0.5 g·L^−1^ day^−1^, which can be defined as 0.0126–0.0633 %*v/v* in terms of percent volume concentration of a solution. This results in minute refractive index changes within the volume of the chemical solution. Before characterizing the sensor for measuring the initial rate of bioethanol production using microalgae in ethanol-producing microalgal mixtures, experiments were performed for ethanol–water solutions with various concentration ranges of ethanol, i.e., 1 to 10 %*v/v*, 0.1 to 1 %*v/v*, and 0.00633 to 0.0633 %*v/v*. An Abbe Refractometer has an RI resolution limit of 10^−4^ RIU which is not sufficient for the intended ethanol–water solutions at very low ethanol concentration ranges. Hence, to determine the refractive indices of the tested ethanol concentration ranges, a mathematical estimation of the refractive indices was performed using a prediction formula, i.e., the Lorentz–Lorenz equation, as demonstrated in previous work by the authors of this article [21]. Table 2 lists the calculated RI values for the ethanol water solutions from 0.00633 %*v/v* to 10 %*v/v* ethanol.

The sensitivity *S* of the sensor can be defined as:

(4) $$S\;\left(\%/\mathrm{RIU}\right)=\frac{\Delta T\%}{\Delta n}$$

where ΔT% is the shift in transmittance % at a changing refractive index of the solution Δn. The resolution *R* of the sensor is expressed as the ratio of the sensor output, i.e., T% at the respective sensor input, i.e., refractive index *n*, to the sensitivity *S* as defined by [30]:

(5) $$R\;\left(\mathrm{RIU}\right)=\frac{T\%}{S}$$

The limit of detection (LOD) of the sensor is determined by:

(6) $$\mathrm{LOD}\;\left(\mathrm{RIU}\right)=\frac{3\sigma }{S}$$

where *σ* represents the standard deviation in the sensor’s response and *S* is the sensitivity of the sensor calculated using Equation (4). The LOD of the sensor was calculated from the measured data samples for the lowest RI or the corresponding ethanol concentration solutions/mixtures.

**Figure 11:**
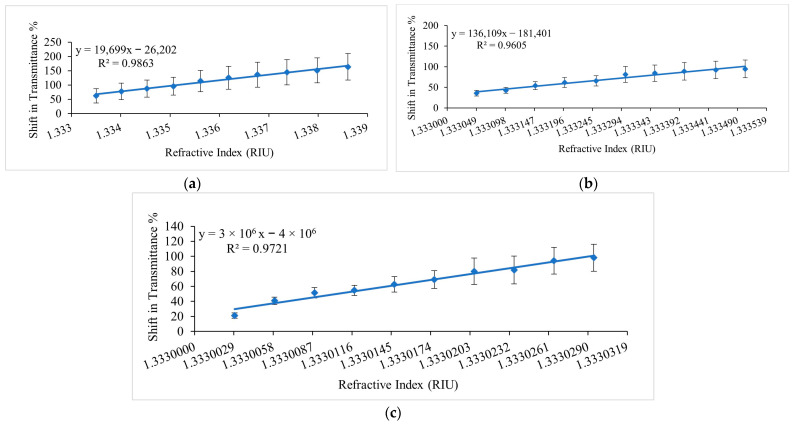
Average of repeated measurements in ethanol–water solutions at 691.45 nm presented as the shift in transmittance % of the sensor versus RI values of different ethanol concentration ranges. (**a**) 1 to 10 %*v/v* ethanol concentration range, (**b**) 0.1 to 1 %*v/v* ethanol concentration range, (**c**) 0.00633 to 0.0633 %*v/v* ethanol concentration range.

The sensor’s response to ethanol concentration was analysed for ethanol–water solutions with three different ranges of ethanol concentrations. A beaker of 100 mL distilled water was set in the ice bath to maintain the temperature and ethanol was added to the solution step by step according to the amount calculated for each range and the corresponding spectrum was recorded in real-time. The measurements were repeated six times and the graphs for the average shift in transmittance % at the corresponding RIs at a wavelength of 691.45 nm are presented in Figure 11. Figure 11a presents the changes in transmittance (percentage) as a function of the changing RI for 1 to 10 %*v/v* ethanol concentration in water. An increase in ethanol concentration modulated the sensor’s response with an increase in transmittance percent as described in Section 3 (sensor calibration). In this range of ethanol concentrations, the linear fitting results predicted a sensitivity of 19,699 %/RIU with an R^2^ of 0.9863. Figure 11b depicts the response of the sensor for 0.1 to 1 %*v/v* ethanol concentrations in water as a function of the corresponding RI values as mentioned in Table 2. For 0.1 to 1 %*v/v* ethanol concentrations in water, the sensor exhibited a sensitivity of 131,609 %/RIU with an R^2^ of 0.9605. Figure 11c demonstrates the sensor’s response to ultra-low ethanol concentration changes in water, i.e., 0.00633 to 0.0633 %*v/v*, which showed that the lower detection limit is even below the initial rate of bioethanol production using microalgae. The highest achieved sensitivity of the sensor in ethanol–water solutions was 2,681,372.9 %/RIU, equivalent to a 3 × 10^6^ %/RIU with an R^2^ of 0.9721. The resolution and limit of detection at the highest achieved sensitivity was 7.9 × 10^−6^ RIU and 9.7 × 10^−6^ RIU, respectively. These measurements essentially cover the whole range of ethanol concentrations in ethanol–water solutions, which makes the sensor design a versatile solution for many ethanol-sensing applications and combined with its high resolution, this sensor design is a promising candidate for bioethanol production applications.

### 4.2. Analysis of Ethanol Sensing in Ethanol-Producing Microalgal Mixtures

**Figure 12:**
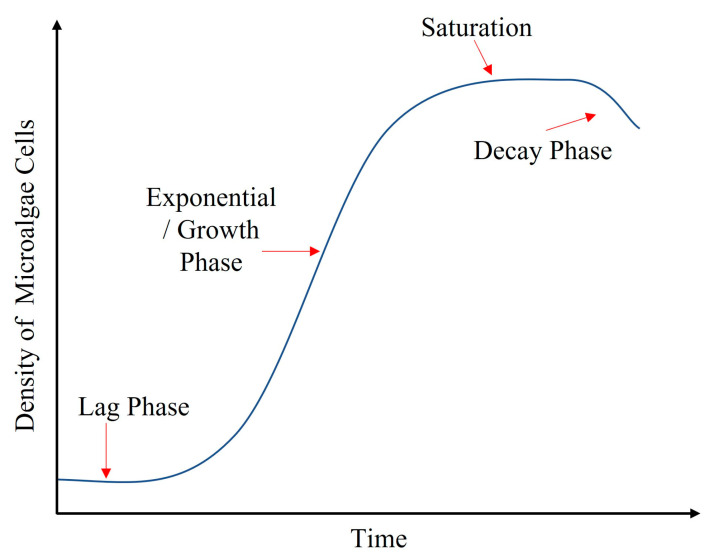
Life cycle of microalgae cells.

After achieving significant results for ethanol sensing in ethanol–water solutions, the proposed LPG corrugated surface, balloon-shaped heterocore-structured POF sensor was characterized for real-time ethanol sensing under actual culturing and production conditions, i.e., ethanol-producing microalgal mixtures. As explained in Section 3, the microalgal mixtures used for bioethanol production are a combination of BG11 broth, distilled water, and the model Cyanobacterium, *Synechocystis*. Before experimentation with microalgal mixtures, the following factors related to biological variations of the microalgal cells need to be considered:

1. Variation in microalgae concentration that occurs with the changing phases of the life cycle.
2. The life cycle of microalgae cells in the mixture is explained in Figure 12 and is a change in the density of microalgae cells where:
  a. The lag phase is when the organisms adapt to the mixture’s environment and surroundings, i.e., pH, temperature, nutrients, and light.
  b. The exponential/growth stage is the phase of maximum growth where maximum cell division occurs, increasing the green pigment in the mixture.
  c. The death/decay phase is when microalgae cells start dying off and cause a change in the optimal pH of the mixture.
3. Excreted products from the microalgae cells.
4. The amount of light-harvesting pigments produced during growth.

These varying biological factors of microalgae cells modify the properties of the mixture and could potentially interfere with the sensor response at different life cycle phases of the microalgal cells. Hence, for optimal consistency of microalgae mixtures, we chose to utilize cultures on the same day the mixtures were prepared. At this stage, the growth process of the microalgae cells is not rapid, and storing the mixture in the dark at refrigerator temperatures further slows down their growth. Therefore, all experiments were performed on the same day that the mixtures were prepared. Being a complex chemical mixture, it was somewhat complicated to determine the RI of the mixture using available laboratory-based equipment or the Lorentz–Lorenz equation that was used for the binary ethanol–water solutions. Hence, for the analysis of the proposed sensor for ethanol sensing in microalgal mixtures, the RI values of the ethanol–water solutions were used as a reference, and ethanol concentration was also used for analysis in terms of %volume/volume (%*v/v*).

**Figure 13:**
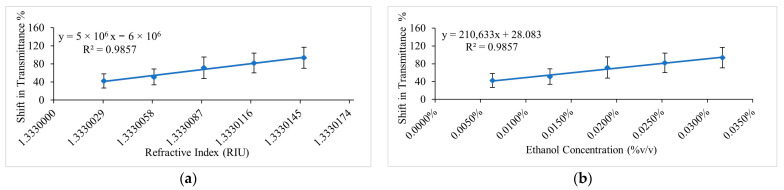
Average of repeated measurements in ethanol–microalgae mixtures at 694 nm for the range of 0.00633 to 0.03165 %*v/v* ethanol concentration. (**a**) Sensor’s response as the shift in transmittance % versus ethanol–water RI values that were used as a reference. (**b**) Sensor’s response as the shift in transmittance % versus %*v/v* ethanol concentration in microalgae mixtures.

For the real-time measurements, the same experimental setup as the ethanol-water solutions analysis was used. In this case, experiments were performed over the range of 0.00633 to 0.03165 %*v/v* ethanol concentration which represents the initial bioethanol production rate using the cyanobacterium. The measurements were repeated five times to determine the repeatability of the sensor’s response to microalgal mixtures. Figure 13 presents the average of repeated sensor measurements for the 0.00633 to 0.03165 %*v/v* ethanol concentration range in the microalgae mixtures at a wavelength of 694 nm. Figure 13a shows the average of repeated measurements as the shift in transmittance % versus RI of the ethanol–water solutions as a reference for the ethanol–microalgae mixtures. Figure 13b shows the average of repeated measurements as the shift in transmittance % versus ethanol concentration in the microalgae mixtures. A linear regression analysis of the averaged measurements produced an R^2^ of 0.9857. The sensor had a sensitivity of 4,665,053.7 %/RIU which is equivalent to 5 × 10^6^ %/RIU and 210,632.8 %T/%*v/v* (% Transmittance/% volume/volume) as calculated from the measurements in terms of RI and % volume/volume ethanol concentration in the microalgae mixtures, respectively. In terms of RIU, the sensor demonstrated a resolution and LOD of 9.4 × 10^−6^ RIU and 2.3 × 10^−5^ RIU, respectively. In terms of ethanol concentration in %*v/v*, the sensor demonstrated a resolution and LOD of 2 × 10^−4^ %*v/v* and 4.9 × 10^−4^ %*v/v*, respectively.

### 4.3. Sensor’s Response and Recovery Time Analysis

**Figure 14:**
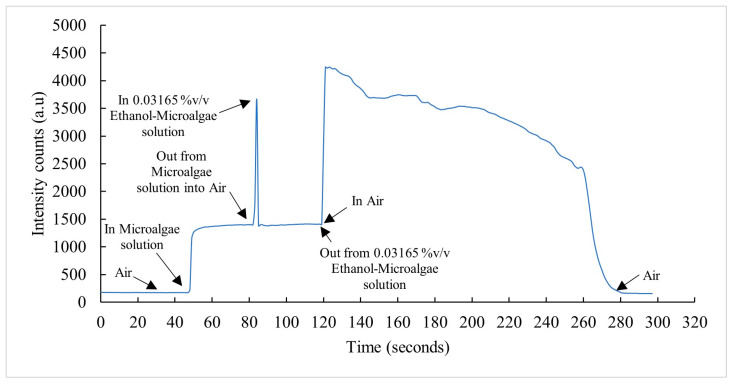
Real-time response of sensor at different stages of measurement as intensity counts at 694 nm versus time in seconds.

**Figure 15:**
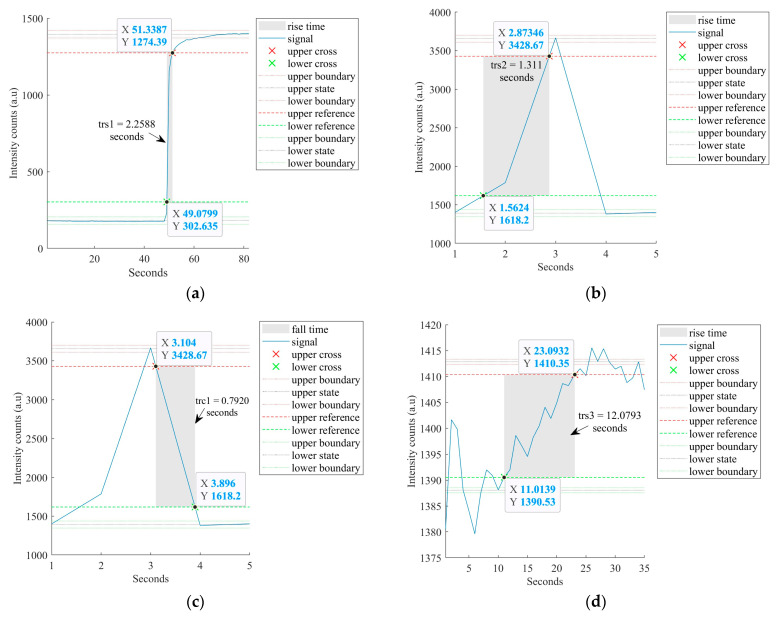

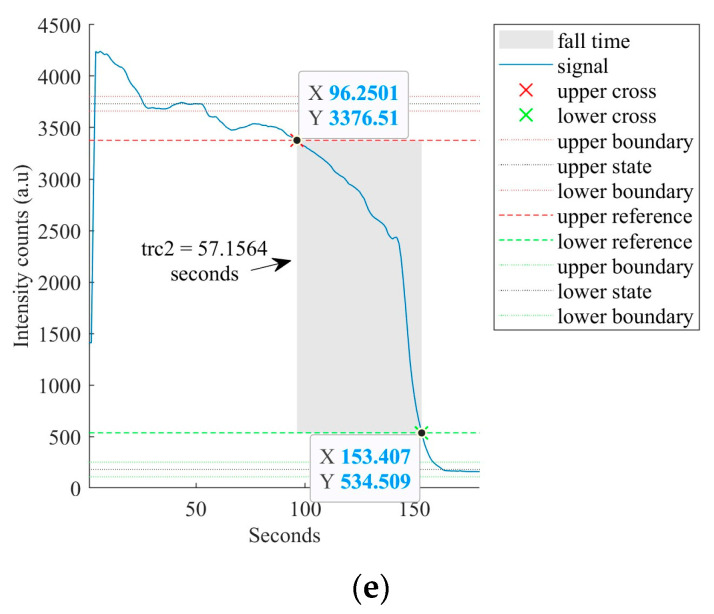
Real-time analysis of sensor’s response and recovery time in the ethanol–microalgae solution presented in Figure 13. (**a**) Analysis for trs1—response time from air to microalgae solution. (**b**) Analysis of trs2—rise time while the sensor is withdrawn from microalgae solution to the air. (**c**) Analysis of trc1—recovery/fall time from air to the 0.03165% ethanol–microalgae solution. (**d**) Analysis of trs3—response time to reach 90% response when the sensor is inserted in 0.03165% ethanol–microalgae solution. (**e**) Analysis of trc2—recovery time from 0.03165% ethanol–microalgae solution to air.

A response and recovery time analysis was performed for the sensor of this investigation for ethanol measurements in the presence of microalgae. The response and recovery times were measured by acquiring the real-time response of the sensor at different states of measurement. The response/rise time corresponds to the time taken by the sensor output to transition from 10% to 90% of the full change in the given state. The recovery/fall time represents the sensor’s output to transition from 90% to 10% of the signal in the given instance. The results corresponding to the time-resolved measurements are shown in Figure 14 and Figure 15.

The sensor was inserted into the microalgae suspension and the response/rise time (trs1) was measured. To determine the sensor’s response time for the ethanol–microalgae suspension, the sensor was removed and quickly inserted into an ethanol–microalgae mixture with 0.03165 %*v/v* ethanol. While withdrawing the sensor from the microalgae mixture into the air and then inserting it into an ethanol–microalgae suspension, a peak was developed in the time response demonstrating the sensor’s rapid response to the change in its environment. The rise time (trs2) of the sensor in the air and fall time (trc1) of the sensor from the air to the ethanol–microalgae mixture were measured. The sensor’s response time (trs3) to transition from 10% to 90% of the intensity in the ethanol–microalgae suspension was determined. To measure the sensor’s recovery/fall time (trc2) from the ethanol–microalgae mixture to air, the sensor was taken out of the mixture.

Figure 14 presents the sensor’s response of the real-time measurement described above at a wavelength 694 nm and Figure 15a–e shows the analysis of each instance of the real-time measurement, determining trs1, trs2, trc1, trs3, and trc2. The signal processing of this real-time calculated response times, i.e., trs1, trs2, and trs3 were 2.2588 s, 1.1311 s, and 12.0793 s, respectively, which represents the sensor’s fast response time from air to the microalgae mixture and from the microalgae mixture to air and fast adaptation to the ethanol–microalgae mixture from the air while changing the mixture beakers. The calculated recovery time, i.e., trc1 and trc2, were 0.7920 s and 57.1564 s, respectively. The higher recovery time trc2 suggests the clinging of the ethanol–microalgae mixture on the sensor.

## 5. Discussion

Table 3 summarizes the measured sensor parameters of the corrugated LPG surface, balloon-shaped heterocore-structured POF sensor, representing excellent sensitivity, high resolution, and LOD in the ethanol–water solutions and ethanol–microalgae mixtures.

Figure 14 and Figure 15 detail the real-time response of the sensor to various instances in the measurement process in ethanol–microalgae mixtures and analyses the response and recovery time at each instance, demonstrating fast response and recovery times. Figure 11 shows the repeatability of the measurements of the sensor of this investigation in ethanol–water solutions at different ethanol concentrations and Figure 13 shows the repeatability of the measurements in ethanol–microalgae suspensions at ethanol concentrations corresponding to the initial rate of bioethanol production using microalgae.

The sensor demonstrated high sensitivity and high resolution corresponding to the initial rate of bioethanol production using microalgae in the water and in the actual culturing and production conditions as detailed in Table 3. The experiments were performed in a laboratory setup in a dark room at low temperatures. For the industrial setting, temperature and ambient light interference need to be precluded or compensated for. For temperature interference, a possible solution could be to determine the sensor response to the potential temperature changes and incorporate localized temperature compensation into the sensing system, e.g., using a standard thermocouple or optical fibre Bragg grating (FBG). For ambient light interference, the sensor could be enclosed in a darkened enclosure with an opening for the mixture to pass through it when the sensor is operating in the industrial bioreactor.

Further optimization of the physical parameters of the sensor presented in this investigation (i.e., the diameters of the fibre cores, period of the grating, radius, and length of the balloon structure) may enhance the sensor’s performance. The heterocore structure presented in this design has been adapted to reflect the classical configuration of the MSM heterocore structure. The large core fibre (LCF) was chosen to have a 1000 µm diameter POF so as to have the maximum possible light transmission prior to coupling to the small core fibre (SCF). As per the literature, small core POFs imprinted with an LPG demonstrate higher sensitivity in comparison to the case of imprinting in a large core POFs [25,31]. The diameter of the SCF and grating period of the LPG used in this investigation were 250 µm and 100 µm, respectively. SCFs with diameters smaller than 250 µm may further enhance the sensitivity of the sensor but may also introduce mechanical robustness challenges. The grating period was determined by the pitch of the thread rod mould used for LPG imprinting but can be optimized with a mould of a different period (pitch) for further performance enhancement. This would have to be specifically machined to generate this value.

The length and radius of the balloon structure were optimized with the objective of efficient coupling of the LPG-inscribed, balloon-shaped SCF with the LCFs using the in-house designed mechanical jig described earlier in this article. Both ends of the SCF were inserted through blunt needles to couple with the LCFs in a way that maintains an optimal radius (as small as possible) ensuring good light coupling with the LCFs and mechanical robustness of the balloon structure of the LPG-inscribed SCF. The length of the balloon structure is dependent on the radius of the balloon shape. The achieved dimensions of the sensor design successfully demonstrated the proof of concept and exhibited the extremely high sensitivity required for the real-time measurement of the ultra-low concentrations of ethanol in microalgal bioethanol production applications. Nevertheless, the radius and length of the balloon structure can be changed by using different lengths of SCF and this may also allow further enhancement of the sensor’s performance. The experimental and/or numerical/simulation analysis to determine the effect of different grating periods, fibre core diameters, radius, and length of the balloon-shaped structure specific to the POF-based heterocore structure design presented in this investigation is the subject of ongoing and future research.

## 6. Conclusions

A novel POF-based corrugated LPG surface balloon-shaped heterocore-structured sensor has been successfully demonstrated for the application of microalgal bioethanol production using the model cyanobacterium, *Synechocystis*. The heterocore structure of the sensor was developed using a large core—small core—large core POF configuration. The small core fibre (SCF), i.e., the sensing region, was modified by polishing, insertion of a mechanically pressed LPG, and macro bending in order to enhance the measurement sensitivity. The sensor is based on the evanescent field interaction at the modified SCF–liquid boundary. The sensor was characterized in real-time for different ethanol concentrations in water, i.e., 20 to 80 %*v/v*, 1 to 10 %*v/v*, 0.1 to 1 %*v/v*, and 0.00633 to 0.0633 %*v/v*. It was further characterized for sensing the initial rate of ethanol production in a laboratory-based culturing and production environment, i.e., blue-green microalgae mixtures for ethanol production. The sensor achieved extremely high resolution on the order of 10^−6^ RIU and 10^−4^ %*v/v* coupled with sensitivities of 3 × 10^6^ %/RIU and 210,632.8 %T/%*v/v* (or 5 × 10^6^ %/RIU) in ethanol–water and ethanol–microalgae suspensions, respectively. A response and recovery time analysis was performed in the ethanol–microalgae suspension at different key instances of the real-time measurement, i.e., in the air, in the microalgae suspension, in the ethanol–microalgae suspension, and in the air. The sensor demonstrated a fast response time and a longer recovery time in comparison due to the nature of the mixture containing the microalgae clinging to the sensor surface. In addition to attractive features of ultra-high resolution and high sensitivity, the use of POF and other readily available components means that the sensor is low cost and easy to fabricate, coupled with excellent response times which demonstrate that it can be an instrumental part of industrial ethanol production plants to optimize the production process at the critical initial production stages and hence improve overall yield. Additionally, the work reported in this article showed that the same sensor has a wide measurement range which can also be utilized for other ethanol-sensing and/or RI-sensing applications. Since the sensing mechanism is based on RI sensing, this sensor can be potentially used in a diverse range of applications, e.g., oxygen concentration measurement in hypoxic tumours and biolayer development measurements in antigen–antibody reactions with the inclusion of application-specific bioreceptors and experimental setups.

## Figures and Tables

**Table 1:** Parameters of the POFs.

Fibre Type	Diameter	Thickness of Cladding	RI of Core	RI of Cladding
DB-250	250 µm	-	1.492	1.402
DC-1000	1000 µm	10 µm	1.492	1.402

**Table 2:** RI Values for the ethanol–water solutions.

Ethanol in %Volume/Volume (%*v/v*)	Refractive Index	Change in Refractive Index (Δ*n*)
0	1.333	0
0.00633	1.33300294673272	0.0000029
0.01266	1.33300589267715	0.0000058
0.01899	1.33300883783337	0.0000088
0.02532	1.33301178220152	0.0000117
0.03165	1.33301472578170	0.0000147
0.03798	1.33301766857403	0.0000176
0.04431	1.33302061057863	0.0000206
0.05064	1.33302355179560	0.0000235
0.05697	1.33302649222506	0.0000264
0.0633	1.33302943186713	0.0000294
0.1	1.33304949718124	0.0000494
0.2	1.33309900807336	0.0000990
0.3	1.33314853206077	0.0001485
0.4	1.33319806852963	0.0001980
0.5	1.33325129792262	0.0002512
0.6	1.33330085869122	0.0003008
0.7	1.33335043011084	0.0003504
0.8	1.33340011477828	0.0004001
0.9	1.33345339125821	0.0004533
1.0	1.33350299199426	0.0005029
2.0	1.33401115772126	0.0010111
3.0	1.33453202665564	0.0015320
4.0	1.33506565132369	0.0020656
5.0	1.33561589378843	0.0026158
6.0	1.33618289103629	0.0031828
7.0	1.33677060634193	0.0037706
8.0	1.33736793591301	0.0043679
9.0	1.33797864363640	0.0049786
10	1.33860298681633	0.0056029

**Table 3:** Performance parameters of the corrugated LPG surface balloon-shaped heterocore-structured POF Sensor.

Chemical Solution	Ethanol Concentration (%*v/v*) or RI Values	Sensitivity	Resolution	LOD
Ethanol–Water Solutions	1 to 10 % *v*/*v* or 1.3335 to 1.3386	19,699 %/RIU	7.9 × 10^−6^ RIU	9.7 × 10^−6^ RIU
	0.1 to 1 %*v/v* or 1.333049 to 1.3335	131,609 %/RIU		
	0.00633 % *v*/*v* to 0.0633 %*v/v* or 1.3330029 to 1.333029	3 × 10^6^ %/RIU		
Ethanol–Microalgae Mixtures	0.00633 to 0.03165 %*v/v* or 1.3330029 to 1.3330147 (as a reference from ethanol-water solutions)	210,632.8 %T/%*v/v* and 5 × 10^6^ %/RIU	2 × 10^−4^ %*v/v* and 9.4 × 10^−6^ RIU	4.9 × 10^−4^ %*v/v* and 2.3 × 10^−5^ RIU

## Data Availability

The data is stored privately on a secure hard disk drive of the author and is backed up on a secure Microsoft cloud location. This data was not made public prior to publication.
